# Lost Opportunities for Smoking Cessation Among Adults With Diabetes in Florida (2007) and Maryland (2006)

**Published:** 2011-04-15

**Authors:** Olivia D. Carter-Pokras, Tammie M. Johnson, Lisa A. Bethune, Cong Ye, Lu Chen, Jacquelyn L. Fried, Robert Fiedler

**Affiliations:** Department of Epidemiology and Biostatistics, University of Maryland College Park School of Public Health; University of North Florida, Jacksonville, Florida; University of Maryland College Park, College Park, Maryland; University of Maryland College Park, College Park, Maryland; University of Maryland College Park, College Park, Maryland; University of Maryland Dental School, Baltimore, Maryland; Maryland Department of Health and Mental Hygiene, Baltimore, Maryland

## Abstract

**Introduction:**

Diabetes organizations recommend that people with diabetes should not smoke because of increased risk of diabetes complications. We describe smoking rates and health care service use among adults with diabetes in Florida and Maryland and identify the role of dentists in offering smoking cessation advice and services.

**Methods:**

We analyzed data from 3 state telephone surveys: the 2007 Florida Behavioral Risk Factor Surveillance Survey (n = 39,549), the 2007 Florida Tobacco Callback Survey (n = 3,560), and the 2006 Maryland Adult Tobacco Survey (n = 21,799).

**Results:**

Findings indicated that 15.7% of adults with diabetes in Florida and 11.6% of adults with diabetes in Maryland currently smoke. Current smoking among people with diabetes was associated with age, education, income, and race/ethnicity. Almost all respondents with diabetes who were current smokers in Florida (92.9%) and Maryland (97.7%) had visited a doctor or health care professional in the past year, and less than half had visited a dentist (40.7% and 44.8%, respectively). Both in Florida and Maryland, approximately two-thirds of adults with diabetes who were smokers and had visited a dentist in the past year had not received advice to quit (63.8% and 63.9%, respectively). In contrast, most adults with diabetes who were smokers and had visited a doctor or health care professional had received advice to quit smoking (95.3% and 84.9%, respectively).

**Conclusion:**

Dentists are in a unique position to identify and demonstrate the oral effects of smoking in patients with diabetes. These data support continued smoking cessation training and education of oral health professionals.

## Introduction

Diabetes and smoking play roles in the development of periodontal and other oral diseases (1,2). One out of every 8 adults at least 20 years of age has diabetes (3), and 16.5% of adults with diabetes smoke (4). Diabetes organizations recommend that people with diabetes should not smoke because of increased risk of diabetes complications (5,6). Adults with diabetes who smoke are 20 times more likely to develop periodontal disease than smokers who do not have diabetes (7), and smoking is a well-established risk factor for gingivitis, oral soft tissue changes, delayed wound healing, oral cancer, and root caries (8,9). Because all of these symptoms are clearly visible through inspection of the oral cavity and 59% of adults with diabetes who smoke see a dentist, dentists are in a unique position to urge smoking cessation, especially to patients with diabetes (10). Most dental schools teach smoking prevention and cessation skills (11); however, a substantial number of dentists do not engage in smoking cessation. In a survey of dentists participating in a large national managed care dental plan, 27.2% of offices reported no smoking cessation activities (12).

This study is a result of a collaboration initiated at the 20th National Conference on Chronic Disease Prevention and Control in 2009, when the first 2 authors (O.D.C.-P., T.M.J.) observed similarities between the data from Florida and Maryland despite dissimilarities between the state populations. The large sample sizes of the surveys and similar survey questions and procedures in the 2 states facilitated the comparisons presented in this article. We used data from 3 state telephone surveys to describe smoking prevalence, stage-of-change readiness, health care use, and receipt of smoking cessation advice from health care professionals and dentists among adults with diabetes in Maryland and Florida. We also provide suggestions for enhancing smoking intervention and management for patients with diabetes based on these findings and the literature.

## Methods

This study used data from 3 cross-sectional telephone surveys conducted of adults residing in Florida and Maryland: the 2007 Florida Behavioral Risk Factor Surveillance Survey (FBRFSS), the 2007 Florida Tobacco Callback Survey (FTCS), and the 2006 Maryland Adult Tobacco Survey (MATS). Interview response rates and analytic sample sizes for the 3 telephone surveys were 55.9% for MATS (n = 21,799), 50.8% for FBRSS (n = 39,549), and 43.3% for FTCS (n = 3,560). We provide the exact wording of key questions used regarding diabetes, smoking, health care professional and dentist visits, receipt of smoking cessation advice, and stages of change (Appendix). We obtained prior approval from the institutional review boards in the respective state departments of health.

The BRFSS is an ongoing, cross-sectional, population-based telephone survey of noninstitutionalized adults aged 18 years or older in randomly selected households in the United States and its territories. The BRFSS elicits from respondents information pertaining to disease states, risk factors, preventive health practices, and emerging health issues (in both English and Spanish in Florida). BRFSS uses a multistage, complex sample design that produces cluster-correlated data (13).

The 2007 FBRFSS had 39,549 respondents, including 8,230 current smokers. Current smokers were adults who responded "yes" to the question "Have you smoked at least 100 cigarettes in your entire life?" and "every day" or "some days" to the question "Do you now smoke cigarettes every day, some days, or not at all?" Of the 8,230 current smokers who participated in the BRFSS, 73% agreed to be contacted again for a callback survey, the FTCS. Of those, 28.1% could not be contacted for follow-up, and 1.7% had quit smoking. The remaining 3,560 participated in the FTCS, 43.3% of the original sample. We merged FTCS data by participant sequential number with the 2007 BRFSS data. As a result, the data collected for the FBRFSS were available for each FTCS participant. We re-weighted the data to account for nonresponse and so that the results could be generalized to Florida adult current smokers. After we merged the data, we defined adults with diabetes as those who responded "yes" to the BRFSS question "Have you ever been told by a doctor that you have diabetes?" We categorized those who responded "yes, but female told only during pregnancy," "no," or "no, prediabetes or borderline diabetes" as nondiabetic adults.

The 2006 MATS was a statewide tobacco survey administered using computer-assisted telephone interviewing technology in both English and Spanish. The MATS sampled 290,700 telephone numbers from all noninstitutionalized Maryland adults (aged 18 years or older) residing in telephone-equipped dwellings by using random-digital-dialing. The sampling design created 24 strata for Maryland's 24 political jurisdictions; each political jurisdiction had 2 substrata reflecting the density of telephone numbers. The analysis took into account the survey stratification, giving a total of 48 strata. The MATS conducted 21,799 interviews. We performed data analyses on the cleaned and weighted data set and used analytic weights constructed to allow the data to be generalized to the entire Maryland adult population and by jurisdiction.

We identified adults with diabetes in the MATS by using the question, "Please tell me if you have EVER been told by a doctor or other health professional that you have diabetes." We categorized women diagnosed with diabetes "only during pregnancy" as not having diabetes. We identified current smokers as having answered "yes" to the question "Have you smoked at least 100 cigarettes in your entire life?" and "every day" or "some days" to the question "Do you now smoke cigarettes every day, some days, or not at all?"

We analyzed data by using SAS version 9.1 (SAS Institute, Inc, Cary, NC), and SUDAAN version 9.0 (Research Triangle Institute, Research Triangle Park, North Carolina). The analyses focused on those adults who self-reported previous diagnosis of diabetes. We calculated proportions and 95% confidence intervals, analyzed data from each survey separately, and compared findings. Significance was set at *P* < .05.

## Results

A previous diagnosis of diabetes was reported by 8.8% of Florida adults and 8.4% of Maryland adults. Compared with adults with diabetes in Maryland, adults with diabetes in Florida had a higher rate of being aged at least 65 years, non-Hispanic white, a college graduate, and having an annual household income of less than $25,000 (Table 1). Numbers of respondents were too small for us to be able to provide separate estimates for racial/ethnic groups other than non-Hispanic whites and non-Hispanic blacks.

Among adults with diabetes, 15.7% in Florida and 11.6% in Maryland were current smokers (Table 2). Current smoking rates among adults with diabetes varied by age, education, income, and race/ethnicity. In both Maryland and Florida, the prevalence of current smoking was higher among adults with diabetes aged 18-44 years (17.3% and 25.4%, respectively) compared with adults with diabetes aged 65 years or older (6.3% and 8.6%, respectively). In Maryland, adults with diabetes in the highest income category (≥$50,000) were less likely to be a current smoker than those in the lowest income category (less than $25,000) for the total population and for non-Hispanic whites. Furthermore, adults with diabetes in Maryland with the least education (less than high school diploma) had higher smoking rates than those with the highest education (college graduate). In Florida, non-Hispanic white adults with diabetes and an income of at least $50,000 had higher smoking rates than non-Hispanic black adults with diabetes  and similar incomes.

Non-Hispanic white adults with diabetes who were current smokers in Florida and Maryland were less likely than non-Hispanic blacks to have stopped smoking for at least 1 day during the past 12 months (Table 3). In both states, the most common stage of change for adults with diabetes who smoke (overall and non-Hispanic whites) was precontemplation (not considering a change in behavior). Among adult current smokers with diabetes in Florida, 66.9% of non-Hispanic whites and 90.2% of non-Hispanic blacks reported that they were not ready to quit,  but that they would be successful in quitting.

More than 9 out of 10 adults with diabetes who smoke had seen a health care professional during the previous year in both states (Table 4). (Florida excluded dentists for this question.) However, less than half of adults with diabetes who smoked had seen a dentist in the past year: 44.8% in Maryland and 40.7% in Florida. Among adults with diabetes who smoked and visited a health care professional during the previous year, 95.3% in Florida (excluded dentists) and 84.9% in Maryland had been advised not to smoke. Of adults with diabetes who smoke who did see a dentist, almost two-thirds in both states were not advised by a dentist to stop smoking.

## Discussion

We used survey data from representative samples of adults from Maryland and Florida. This study indicates the need to raise awareness of the importance of visiting a dentist for adults with diabetes, since more than half of adults with diabetes who were smokers in both states had not visited a dentist during the past year. It also indicates the need for dentists to take a more active role in providing smoking cessation advice, since almost two-thirds of adults with diabetes who smoke and had visited a dentist had not received advice to quit smoking.

Less than half of adults with diabetes who smoke in Florida and Maryland had seen a dentist during the previous year. These findings are similar to national estimates from the 2005 BRFSS (47.2%) (4). By actively engaging in measures to address smoking and diabetes in their patient populations, dentists have opportunities to enhance their patients' oral and systemic health (2,14-18).

Although these cross-sectional surveys cannot provide information on trends over time in provision of smoking cessation advice by dentists, previous studies suggest that many dentists do not routinely incorporate smoking cessation into their practice (12,19,20). Dentists' focus on treatment rather than prevention may contribute to the attitude that their role is not to provide smoking cessation advice, and a reorientation of the dentist's self-perception to focus more on prevention may be the change needed to facilitate smoking intervention and management behaviors for patients with diabetes (2). One study found that, although 61.5% of dentists believe that their patients did not expect smoking cessation advice or services from them, 58.5% of patients did expect those services (20). Dentists have previously given reasons for their low assistance rates in smoking cessation, such as lack of training, lack of time, lack of reimbursement, busyness, and fear that patients will not be receptive (19-21).

Training and knowledge of the Agency for Healthcare Research and Quality clinical practice guideline (15) can substantially increase the likelihood that dentists would assist their patients with cessation (14,20,22). According to the American Dental Education Association, approximately 1 out of 5 dental schools does not provide teaching in smoking cessation skills; however, the number of dentists assisting in smoking cessation has increased after providing such training (20). Therefore, more emphasis needs to be placed on providing smoking cessation training for dentists while in dental school and through continuing education (14,19).

Strengths of the study include the use of representative samples of adults from 2 states, the large number of adults with diabetes who were current smokers (more than 1,000), use of survey methods from the Centers for Disease Control and Prevention, similar survey mode (computer-assisted telephone interviewing), and identical wording of key questions (eg, ever smoked, current smoker, dentist visit). Although question wording differences between the 2 states regarding the stage of change readiness, and health professional visits and advice may partly explain observed differences in estimates between the 2 states, sociodemographic patterns are similar within each state.

There were several limitations to this study. Because our analyses drew from previously collected cross-sectional survey data, we were unable to explore additional relevant questions to further focus on the issues under investigation. Our surveys did not specifically ask about receipt of advice from dental hygienists, so the results may underreport receipt of advice by oral health professionals. In addition, our survey questions regarding receipt of advice from doctors or other health professionals did not exclude dentists in Maryland, or other oral health professionals in both states. Despite the overall large number of adults with diabetes in the surveys, we were unable to examine patterns for racial/ethnic groups other than non-Hispanic whites and blacks because of small numbers. Future studies can further examine racial/ethnic disparities in receipt of smoking cessation advice from health professionals and dentists.

Another limitation is the use of self-reported diabetes diagnosis, which is subject to respondents' access to care and health care use and ability to accurately recall and report a diabetes diagnosis. Smoking cessation in patients with undiagnosed diabetes or prediabetes is also relevant; however, we were unable to identify prediabetes from the MATS or undiagnosed diabetes for either state. Inclusion of key questions used in our analyses in future National Health and Nutrition Examination Survey (NHANES) questionnaires (eg, smoking cessation advice/support from health professionals/dentists, stage-of-change readiness) would permit similar analyses for adults with undiagnosed diabetes and/or prediabetes. During 2005-2006, 29.5% of adults at least 20 years of age had prediabetes, and 12.9% had diabetes (39.8% of those cases were undiagnosed) (3). Oral health professionals, through comprehensive health history interviews and oral examinations, may assist in identifying signs of undiagnosed diabetes or prediabetes such as bleeding gingiva, periodontal disease (2), acetone breath, polyphagia, polyusiria, and polydipsia (23).

Overall, we found that the most common stage of change for adults with diabetes who smoke in Florida and Maryland was precontemplation. Developed by Prochaska and DiClemente, the stages-of-change or trans-theoretical model describes the sequential process by which people overcome addiction (24). For smokers with diabetes who are not interested in quitting (precontemplation), dentists can raise patient awareness about the effects of smoking on the oral cavity and the possibility of better treatment results and long-term oral health if they are tobacco-free, and offer future assistance when such patients become interested in quitting (25,26). To help resolve ambivalence among smoker patients with diabetes who are contemplating cessation (contemplation), dentists can also emphasize the benefits of change and inform them of referral sources and pharmacotherapy options that can be used when they are ready to set a quit date. Smokers with diabetes who are ready to quit within the next month and want more help (preparation) can be referred to group or individual counseling programs or telephone helplines.

Our data indicate that adults with diabetes who smoke are not seeking adequate dental care, and when they do seek care, they often are not being advised by the dentist to quit smoking. The literature reveals that barriers to dentist involvement in smoking cessation and diabetes management exist and that additional training in smoking interventions and the management of the patient with diabetes, particularly during formal education, would increase dental involvement. Overt dentist endorsement of smoking interventions, in combination with the direct delivery of these services, also is needed because these interventions can be rendered by the dental hygienist. Both the dental community and patients with diabetes who smoke will benefit from more research on the dentists' smoking cessation interventions for patients with diabetes.

## Figures and Tables

**Table 1:** Sociodemographic Characteristics of Adults With Diabetes in Maryland (2006) and Florida (2007)^a^

Characteristic	**Maryland (n = 2,335), % (95% CI)**	**Florida (n = 4,947), % (95% CI)^c^**
**Age, y**		
18-44	18.4 (15.3-21.4)	12.5 (10.0-15.6)
45-64	46.1 (42.8-49.5)	40.5 (37.3-43.7)
≥65	**35.5 (32.4-38.6)**	**47.0 (43.8-50.2)**
**Sex**		
Men	49.6 (46.2-52.9)	52.0 (48.8-55.2)
Women	50.4 (47.1-53.8)	48.0 (44.8-51.2)
**Race/ethnicity**		
Non-Hispanic white	**56.8 (53.7-59.8)**	**70.9 (67.4-74.1)**
Non-Hispanic black	**34.3 (31.3-37.2)**	**14.1 (12.0-16.6)**
**Education**		
<High school diploma	13.3 (11.0-15.6)	16.1 (13.9-18.6)
High school graduate/some college	**52.9 (49.5-56.3)**	**30.9 (27.9-34.1)**
College graduate	**33.8 (30.6-37.0)**	**53.0 (49.7-56.2)**
**Annual income, $**		
<25,000	**26.6 (23.4-29.7)**	**37.9 (34.6-41.4)**
25,000-49,999	25.1 (22.0-28.2)	29.4 (26.6-32.4)
≥50,000	**48.3 (44.7-52.0)**	**32.7 (29.4-36.1)**
**Married or partner**		
Yes	58.6 (55.3-61.8)	61.2 (56.9-63.3)
No	41.4 (38.2-44.7)	39.9 (36.7-41.1)

Abbreviation: CI, confidence interval.

a Comparison is made between Maryland and Florida for every category. Numbers are bolded where differences are significant (P < .05). Data sources: 2007 Florida Behavioral Risk Factor Surveillance Survey, 2007 Florida Tobacco Callback Survey, and 2006 Maryland Adult Tobacco Survey.

**Table 2:** Smoking Rates Among Adults With Diabetes by State, Race/Ethnicity, and Sociodemographic Characteristics^a^

**Sociodemographic Characteristics**	**Maryland**			**Florida**		

	**Total (n = 2,325), % (95 % CI) **	**Non-Hispanic White (n = 1,682), % (95% CI)**	**Non-Hispanic Black (n = 486), % (95% CI)**	**Total (n = 4,947), % (95% CI)**	**Non-Hispanic White (n = 3,758), % (95% CI)**	**Non-Hispanic Black (n = 614), % (95% CI)**
All	11.6 (9.5-13.8)	11.6 (8.8-14.4)	13.0 (8.7-17.3)	15.7 (13.5-18.2)	15.7 (13.2-18.5)	16.6 (10.2-25.9)
**Age, y**						
18-44	**17.3 (10.2-24.4)**	21.5 (11.2-31.9)	21.2 (6.7-35.7)	**25.4 (17.1-36.0)**	**27.3 (16.7-41.3)**	**21.1 (9.7-45.5)**
45-64	13.6 (10.2-17.1)	12.4 (8.1-16.7)	14.8 (8.6-20.9)	20.9 (17.1-25.2)	24.4 (19.6-30.1)	13.7 (8.0-22.3)
≥65	**6.3 (4.1-8.4)**	6.8 (3.9-9.6)	5.8 (1.9-9.8)	**8.6 (6.3-11.7)**	**6.4 (4.9-8.3)**	**18.8 (8.0-38.1)**
**Sex**						
Men	12.6 (8.9-16.3)	11.9 (7.3-16.5)	14.6 (6.8-22.4)	17.0 (13.7-21.0)	17.1 (13.3-21.8)	16.6 (7.2-33.7)
Women	10.7 (8.3-13.0)	11.0 (8.0-14.0)	12.2 (7.5-16.9)	14.3 (11.6-17.5)	13.9 (11.3-17.1)	16.6 (9.0-28.7)
**Education**						
<High School diploma	**19.1 (11.3-26.9)**	18.0 (8.4-27.7)	23.4 (10.0-36.7)	22.3 (15.2-31.3)	22.0 (13.1-34.5)	26.1 (11.8-48.4)
High school graduate/some college	12.4 (9.5-15.3)	13.4 (9.6-17.2)	12.1 (6.8-17.5)	14.4 (11.1-18.6)	14.9 (11.0-19.9)	12.3 (6.6-21.8)
College graduate	**7.7 (4.1-11.3)**	6.7 (2.2-11.2)	8.4 (1.0-15.9)	14.7 (12.0-18.0)	15.2 (13.0-19.0)	12.0 (5.5-24.2)
**Annual income, $**						
<25,000	**17.3 (11.9-22.6)**	**22.8 (13.8-31.9)**	12.2 (6.8-17.6)	19.4 (15.3-24.4)	18.6 (14.2-24.0)	18.5 (9.3-33.7)
25,000-49,999	13.0 (8.0-18.0)	10.3 (5.3-15.3)	20.1 (8.5-31.7)	16.6 (12.7-21.5)	17.1 (12.4-23.0)	13.5 (5.4-29.8)
≥50,000	**7.3 (4.3-10.3)**	**8.1 (4.6-11.7)**	6.2 (0-12.8)	11.9 (8.4-16.6)	**14.2 (10.0-19.9)**	**2.2 (0.5-9.5)**
**Married or partner**						
Yes	9.1 (6.5-11.7)	8.6 (5.8-11.3)	11.3 (4.7-17.9)	13.5 (10.9-16.5)	14.0 (11.0-17.6)	8.3 (3.9-16.9)
No	15.5 (11.7-19.2)	17.1 (11.3-22.8)	15.0 (9.3-20.6)	19.2 (15.3-23.8)	18.9 (14.6-24.0)	22.9 (12.9-37.1)

Abbreviation: CI, confidence interval.

a Comparison is made within each state by sociodemographic characteristics. Numbers are bolded where differences are significant (*P* < .05). Data sources: 2007 Florida Behavioral Risk Factor Surveillance Survey, 2007 Florida Tobacco Callback Survey, and 2006 Maryland Adult Tobacco Survey.

**Table 3:** Quit History, Stage of Change, and Self-Perceived Success for Quitting Among Diabetic Current Smokers, by State and Race/Ethnicity^a^

**Characteristic**	**Maryland**			**Florida**		

	**Total (n = 270), % (95% CI)**	Non-Hispanic** White (n = 183), % (95% CI) **	**Non-Hispanic Black (n = 69), % (95% CI) **	**Total (n = 788), % (95% CI) **	**Non-Hispanic White (n = 613), % (95% CI) **	**Non-Hispanic Black (n = 82), % (95% CI) **
**Stopped smoking for at least 1 day in the past 12 months**						
Total	46.8 (36.6-57.1)	**35.0 (25.9-44.0)**	**66.6 (47.2-86.0)**	56.5 (48.3-64.4)	**47.6 (38.4-56.9)**	**81.3 (60.0-92.6)**
Men	48.3 (31.2-65.5)	35.7 (20.8-50.6)	76.3 (45.4-100.0)	51.6 (39.8-63.2)	42.5 (29.9-56.2)	79.9 (46.4-94.8)
Women	45.1 (32.4-57.7)	34.0 (20.7-47.2)	58.0 (36.1-79.8)	62.9 (52.8-72.0)	55.1 (44.5-65.2)	82.1 (51.7-95.1)
**Stage of change^b^**						
Precontemplation	51.9 (42.4-61.4)	61.1 (51.6-70.7)	37.0 (17.8-56.2)	39.8 (34.7-45.1)	43.6 (37.9-49.5)	25.8 (14.0-42.8)
Contemplation	20.4 (12.8-28.0)	15.8 (10.4-21.2)	29.5 (10.6-48.5)	32.5 (27.7-37.8)	33.4 (28.2-39.1)	27.9 (14.1-47.8)
Preparation	27.7 (18.2-37.2)	23.1 (14.1-32.0)	33.5 (12.1-54.9)	27.7 (23.1-32.8)	**23.0 (18.4-28.4)**	**46.2 (29.8-63.6)**
Not ready to quit but thought I would be successful in quitting	72.7 (63.4-82.0)	66.8 (54.5-79.0)	81.4 (66.0-96.8)	72.0 (67.1-76.4)	**66.9 (61.2-72.1)**	**90.2 (75.8-96.5)**

Abbreviation: NH, Non-Hispanic.

a Comparison is made between Non-Hispanic Whites and Non-Hispanic Blacks within each state. Numbers are bolded where differences are significant. Data sources: 2007 Florida Behavioral Risk Factor Surveillance Survey, 2007 Florida Tobacco Callback Survey, and 2006 Maryland Adult Tobacco Survey.

b Smokers with diabetes who are not interested in quitting (precontemplation), smokers with diabetes who are contemplating cessation (contemplation), smokers with diabetes who are ready to quit within the next month and want more help (preparation).

**Table 4:** Health Professional and Dental Visits and Receipt of Advice to Quit Smoking From Health Professionals and Dentists Among Smokers With Diabetes by State and Race/Ethnicity^a^^,^ b

Characteristic	Maryland (n = 270), % (95% CI)	Florida (n = 588), % (95% CI)
**Visited a doctor or health professional in the past year**		
Total	**97.7 (95.5-100.0)**	**92.9 (89.5-95.2)**
Non-Hispanic white	98.3 (95.4-100.0)	93.9 (90.5-96.1)
Non-Hispanic black	97.9 (95.2-100.0)	92.8 (75.6-98.2)
**Among those who visited a health professional, percentage who were advised not to smoke**		
Total	84.9 (76.7-93.2)	95.3 (92.4-97.2)
Non-Hispanic white	90.2 (84.8-95.7)	94.6 (91.0-96.9)
Non-Hispanic black	78.5 (56.4-100.0)	97.2 (82.2-99.6)
**Saw a dentist in the past year**		
Total	44.8 (34.2-55.5)	40.7 (35.7-45.9)
Non-Hispanic white	48.3 (35.2-61.3)	43.5 (37.9-49.2)
Non-Hispanic black	39.5 (20.7-58.3)	26.8 (15.1-43.0)
**Among those who saw a dentist, percentage who were advised not to smoke**		
Total	36.1 (24.3-47.9)	36.2 (28.7-44.5)
Non-Hispanic white	23.5 (14.4-32.6)	37.5 (29.5-46.2)
Non-Hispanic black	59.4 (31.7-87.0)	38.4 (14.9-68.8)

a Comparison is made between Maryland and Florida for each category. Numbers are bolded where differences are significant (*P* < .05).

b Data sources: 2007 Florida Behavioral Risk Factor Surveillance Survey, 2007 Florida Tobacco Callback Survey, and 2006 Maryland Adult Tobacco Survey.

## Appendix. Questions Used Regarding Diabetes, Smoking, Health Professional and Dentist Visits and Advice, and Stages of Change: 2006 Maryland Adult Tobacco Survey (MATS), 2007 Florida Behavioral Risk Factor Surveillance Survey (FBRFSS), and 2007 Florida Tobacco Callback Survey (FTCS)

**Table:**

**Topic/Survey**	**Question**
**Previous diabetes diagnosis**	
2006 MATS	I am going to read a list of medical conditions that many people have. After each one, please tell me if you have EVER been told by a doctor or other health professional that you have that condition. Diabetes? 1. Yes 2. No 3. Only during pregnancy 777. Don't know/not sure 999. Refused
2007 FBRFSS	Have you ever been told by a doctor that you have diabetes? 1. Yes 2. Yes, but female told only during pregnancy 3. No 4. No, pre-diabetes or borderline diabetes 7. Don't know/not sure 9. Refused
**Ever smoked**	
2006 MATS	Have you smoked at least 100 cigarettes in your entire life? [Note: 100 cigarettes is equal to 5 packs] 1. Yes 2. No 777. Don't know/not sure 999. Refused
2007 FBRFSS	Have you smoked at least 100 cigarettes in your entire life? 1. Yes 2. No (Skip "Current Smoker" Question) 7. Don't know/not sure 9. Refused
**Current smoker**	
2006 MATS	Do you now smoke cigarettes every day, some days, or not at all? 1. Every day 2. Some days 3. Not at all 777. Don't know/not sure 999. Refused
2007 FBRFSS	Do you now smoke cigarettes every day, some days, or not at all? 1. Every day 2. Some days 3. Not at all (not contacted for FTCS) 7. Don't know/not sure 9. Refused
**Visit health professional**	
2006 MATS	In the past 12 months have you gone to a doctor or other health professional for a check-up or medical treatment? 1. Yes 2. No 777. Don't Know/Not Sure 999. Refused
2007 FTCS	In the PAST 12 MONTHS, have you seen a doctor, nurse, or other health professional other than a dentist to get any kind of care for yourself? 1. Yes 2. No (Skip "Health professional advice" question) 7. Don't know/Not sure 9. Refused
**Visit dentist**	
2006 MATS	In the past 12 months, have you seen a dentist? 1. Yes 2. No 777. Don't Know/Not Sure 999. Refused
2007 FTCS	In the PAST 12 MONTHS have you seen a dentist? 1. Yes 2. No (Skip "Dentist Advice" Question) 7. Don't know/Not sure 9. Refused
**Health professional advice**	
2006 MATS	Has a doctor, dentist, or other health professional EVER advised you to quit smoking? 1. Yes 2. No 777. Don't know/not sure 999. Refused (*If Yes*) During the past 12 months, did any doctor, nurse, or other health professional ADVISE YOU not to smoke? 1.Yes 2. No 777. Don't know/not sure 999. Refused
2007 FTCS	In the PAST 12 MONTHS, did any doctor, nurse, or other health professional other than a dentist advise you not to smoke? 1. Yes 2. No 7. Don't know/Not sure 9. Refused
**Dentist advice**	
2006 MATS	(*If visited dentist in past 12 months*) During the past 12 months, did your dentist ADVISE YOU not to smoke? 1. Yes 2. No 777. Don't know/not sure 999. Refused
2007 FTCS	During the PAST 12 MONTHS, did any dentist ADVISE you to stop smoking? 1. Yes 2. No 7. Don't know/not sure 9. Refused
**Stages of change**	
2006 MATS	Are you seriously planning to quit smoking cigarettes 1. Within the next 30 days 2. Within the next 3 months 3. Within the next 6 months 4. Within the next 12 months 5. Within the next 5 years 6. Sometime after 5 years 8. I am not planning on quitting 777. Don't know/not sure 999. Refused
2007 FTCS	Are you seriously considering stopping smoking within the next six months? 1. Yes 2. No 7. Don't know/not sure 9. Refused Are you planning to stop smoking within the next 30 days? 1. Yes 2. No 7. Don't know/Not sure 9. Refused
